# Breast Support Garments are Ineffective at Reducing Breast Motion During an Aqua Aerobics Jumping Exercise

**DOI:** 10.1515/hukin-2015-0033

**Published:** 2015-07-10

**Authors:** Chris Mills, Bessie Ayres, Joanna Scurr

**Affiliations:** 1 Department of Sport and Exercise Science, Spinnaker Building, University of Portsmouth, PO1 2ER.

**Keywords:** water, sports bra, biomechanics, kinematics

## Abstract

The buoyant forces of water during aquatic exercise may provide a form of ‘natural’ breast support and help to minimise breast motion and alleviate exercise induced breast pain. Six larger-breasted females performed standing vertical land and water-based jumps, whilst wearing three breast support conditions. Underwater video cameras recorded the motion of the trunk and right breast. Trunk and relative breast kinematics were calculated as well as exercised induced breast pain scores. Key results showed that the swimsuit and sports bra were able to significantly reduce the superioinferior breast range of motion by 0.04 and 0.05 m, respectively, and peak velocity by 0.23 and 0.33 m/s, respectively, during land-based jumping when compared to the bare-breasted condition, but were ineffective at reducing breast kinematics during water-based jumping. Furthermore, the magnitude of the swimsuit superioinferior breast range of motion during water-based jumping was significantly greater than land-based jumping (0.13 m and 0.06 m), yet there were no significant differences in exercise induced breast pain, thus contradicting previously published relationships between these parameters on land. Furthermore, the addition of an external breast support garment was able to reduce breast kinematics on land but not in water, suggesting the swimsuit and sports bras were ineffective and improvements in swimwear breast support garments may help to reduce excessive breast motion during aqua aerobic jumping exercises.

## Introduction

Physical inactivity and subsequent deconditioning of the cardiovascular and musculoskeletal systems have been shown to negatively affect health and increase the risk of conditions such as cardiovascular disease, obesity and type 2 diabetes (O’Donovan et al., 2010). Exercise prescription is a fundamental practice of doctors and health professionals as a means of the promotion of health / physical well-being. Water-based exercise activities are growing in popularity (Becker, 2009) and have been reported to be as effective as land-based training in terms of improving health (Benelli et al., 2014). The increased density of water and the drag force it creates provide additional resistance to the body and help to improve the physical conditioning of individuals (Colado et al., 2009; Triplett et al., 2009). Aquatic exercise / therapy are also commonly recommended for people who experience pain whilst exercising on land (Ariyoshi et al., 1999; Westby, 2001). It is proposed that the buoyant forces of water offer support, reducing loading and pain in the injured sites (Ariyoshi et al., 1999; Evans et al., 1978; Westby, 2001).

A group of patients who frequently seek advice and treatment for pain are women with larger breasts. When exercising on land, in such activities as aerobics, the jumping component of these fitness exercises can induce breast displacements of 0.187 m vertically, combined with peak breast velocities of 0.931 m/s in breasts that are not adequately supported (Bridgman et al., 2010). Previous biomechanical research has also found a positive relationship between increased exercised induced breast pain and increased breast displacement and velocity (Scurr et al., 2010). If the increased density of water compared with air increases the hydrostatic pressure (Pendergast and Lundgren, 2009), this will push inwards on the rib cage and can have a chest restricting effect (Robertson et al., 1978) which may also provide a form of ‘natural’ breast support during exercise in water, whilst the breasts are submerged. As well as offering support to the breasts, the water may also help to alleviate the exercise induced breast pain experienced by women with larger breasts when they exercise on land. However, it is also possible that additional resistance provided by the water may induce a greater load on the breast as the thorax moves out of the water and the breasts ‘lag’ behind, thus increasing breast displacement and pain.

While the amount of breast displacement and the effect of varying types and levels of breast support have been documented on land (White et al., 2009; Bridgman et al., 2010; Scurr et al., 2010; Zhou et al., 2012), there have been very few studies to date examining breast biomechanics in water. The water may provide a similar ‘external’ support to that of a sports bra, thus leading to a reduction in breast kinematics and pain (McGhee et al., 2007). Additionally, the ‘natural’ support of the water, when combined with an external support garment (i.e. a swimsuit or a sports bra), may provide additional support to the breasts and further decrease exercise induced breast pain in water.

Whilst one research study has investigated breast motion and pain during running on land and in water, most land based exercise classes and conditioning programmes incorporate some form of jumping (step aerobics, circuits). Jumping is also a part of aqua aerobics (Aquatic Exercise Association, 2008); however, during typical water-based exercises the participant is generally chest deep (Aquatic Exercise Association, 2008), therefore, during the execution of a jump in water the breasts often transition from the water into the air and back again. Understanding the movement behaviour of the breasts during a water-based jumping exercise will help inform the requirements of breast support garments in the water environment as well as the suitability of water-based jumping as a means to reduce breast pain for exercise and conditioning programmes that include a jumping component (Kamalakkannan et al., 2010). Therefore, this preliminary study aimed to investigate the effect of breast support on breast kinematics and exercise induced breast pain during water and land-based jumping. Firstly, it was hypothesised that there would be significant differences in breast kinematics between breast support garments during water and land-based jumping. Secondly, there would be no significant differences in the breast range of motion (ROM) and peak breast velocity during water-based jumping compared to land-based jumping. Finally, it was hypothesised that exercised induced pain would be significantly lower during water-based jumping.

## Material and Methods

### Participants

Six large breasted females (UK sizing: 34F, 34F, 30G, 34G, 36FF and 34HH) were recruited for this study (age: 29 ± 7 years; body mass: 78.9 ± 14.9 kg; body height: 1.66 ± 0.05 m). Women with larger breasts were selected as Lorentzen and Lawson (1987) identified that controlling the breast ROM and hence minimising exercise induced breast pain was of most importance in this size range. Participants were pre-menopausal, physically active, had not experienced any surgical procedures to the breasts, and were not pregnant or breast feeding within the last year. Following institutional ethical approval and prior to testing each participant gave written informed consent and completed a health history questionnaire. They also had their blood pressure checked to ensure it was within the institutional guidelines. Participants’ bra size was established by a trained bra fitter and fitted in the sports bra used for testing (using the fit criteria as set out by White and Scurr (2012)). Participant’s swimsuits were sized according to the manufacturer’s guidelines.

### Procedures

Two jumping conditions (land-based and water-based) were completed by each participant. The water-based jumps were completed in a swimming flume (600-T, SwimEx Inc., USA) (water temperature: 30.5°C ± 1°C) and the land-based jumps were completed in the research lab (lab temperature 22°C). For both jumping conditions the participants were filmed using two synchronised underwater cameras (VB5C6 Submersible Colour Camera, Videcon PLC) sampling at 25 Hz with a resolution of 720 by 576 pixels. The two camera views were synchronised using an event synchronisation (light flash) viewed in all cameras. During the water-based jumps the two cameras were placed in front of the participant, one above the water and one below. The same camera orientations and relative positions were used during the land-based jumps. The activity volume was calibrated using a 17 point calibration frame (Sputnik Calibration Frame, Simi Reality Motion Systems) and was part submerged in the water.

Following calibration, water refraction and lens distortion error were corrected for in Simi Motion Analysis software (Version 5.5) using 12 DLT parameters. The underwater filming reconstruction accuracy was assessed using a board covered with markers with 0.1 m separations arranged in a 10 × 10 grid. Sixteen of these markers were digitised in Simi and the reconstructed distances between the markers were compared to the known distances; the average error for the underwater filming was 3 mm.

**Figure 1:**
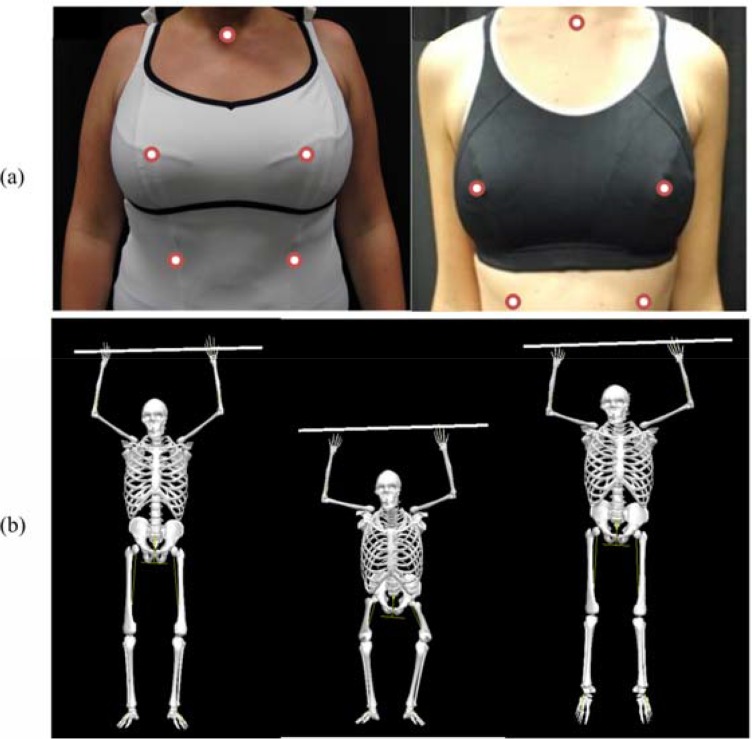
Examples of (a) markers placements and breast support conditions (left = swimsuit, right = sports bra), (b) the jumping technique.

Custom made, fibre optic markers were adhered to the skin using hypoallergenic waterproof tape (under clothing). Markers were attached to landmarks at the sternal notch, the right nipple and the left and right anterior inferior aspect of the 10^th^ ribs (Scurr et al., 2009; 2010; White et al., 2009) (Figure 1a). Before each activity the participant was given three to five minutes to warm-up (running or swimming and jumping) and to familiarise themselves with the equipment and exercise activity. The testing consisted of three maximum effort, continuous, vertical jumps; this was repeated both on land and in water (Figure 1b). During both trials, the participants held a tubular float above their head to keep their arms in a standard position and mimic water aerobics activities (Aquatic Exercise Association, 2008). When water-based jumping, all of the participants began the jumps with their sternal notch at the water’s surface (floor of flume adjusted to standardise water depth), and breasts underneath the water; they then jumped up out of the water and landed with the breasts underneath the water again. Following each trial participants completed numerical analogue pain scales, on which they rated their exercise-induced breast pain on a scale of 0 (no pain) to 10 (painful) (Mason et al., 1999). Each jumping condition was performed in three breast support conditions; bare-breasted, swimsuit (71% Polyamide, 29% Elastane), the best-selling swimsuit for recreational swimmers in the UK and a sports bra (45% Polyester, 44% Polyamide and 11% Elastane), the 2008 best-selling branded sports bra in the UK, allocated in a random order (Figure 1a).

Digital video footage of the jumping conditions was uploaded to Simi and following calibration of the synchronised footage, anatomical markers were manually digitised for each participant, during each jump in each breast support condition. After reconstruction, marker coordinate data were exported into Microsoft Excel. The sternal notch marker (origin) was used to calculate a vertical trunk range of motion by subtracting minima positional coordinates from maxima coordinates during each jump. A trunk reference segment was constructed using the markers on the suprasternal notch and left and right ribs, this was used to convert the motion of the right nipple from the global coordinate system to a local, relative coordinate system enabling independent relative motion of the right nipple to be determined (Scurr et al., 2010). The local coordinate system identified *y* as mediolateral and *z* as superioinferior. Relative breast coordinates were filtered using a 2^nd^ order low-pass Butterworth filter (cut-off frequency of 8 Hz). This cut-off frequency was determined using a customised MatLab programme which enabled the power spectrum and residual analysis of the signal to be analysed (Winter, 1990). Superioinferior and mediolateral relative breast ranges of motion were calculated by subtracting minima positional coordinates from maxima coordinates during each jump (adapted from gait assessment; Scurr et al., 2010). Breast velocity was determined from the differentiated positional data and the absolute peak velocity of the breast identified within each of the three jumps. The mean trunk and breast range of motion and peak breast velocity were calculated from the three trials in each breast support condition in both the water and land-based jumps.

### Statistical Analysis

Trunk and breast kinematics data and exercise induced breast pain scores were statistically analysed using PASW software (Version 18). All data were checked for normality using the Shapiro-Wilk test and were parametric if p > 0.05. Repeated Measures ANOVAs were used when the data were normally distributed and a Friedman test was used for non-parametric data. ANOVAs were followed by post-hoc analysis in the form of multiple paired samples T-tests with a Bonferroni adjustment. Effect sizes (Partial eta squared ‘η^2^’ and / or Cohen’s ‘d’) are reported for significant results to provide an indication of the magnitude of the result. A large effect size was defined as d or η^2^ > 0.8, moderate as between 0.8 and 0.5, and a small effect size defined as < 0.5 (Field, 2009). Qualitative exercise-induced breast pain data were non-parametric; therefore, statistical comparisons were made using a Friedman test, followed by post-hoc Wilcoxon Signed Ranks tests.

## Results

### Vertical Trunk Range of Motion

**Figure 2:**
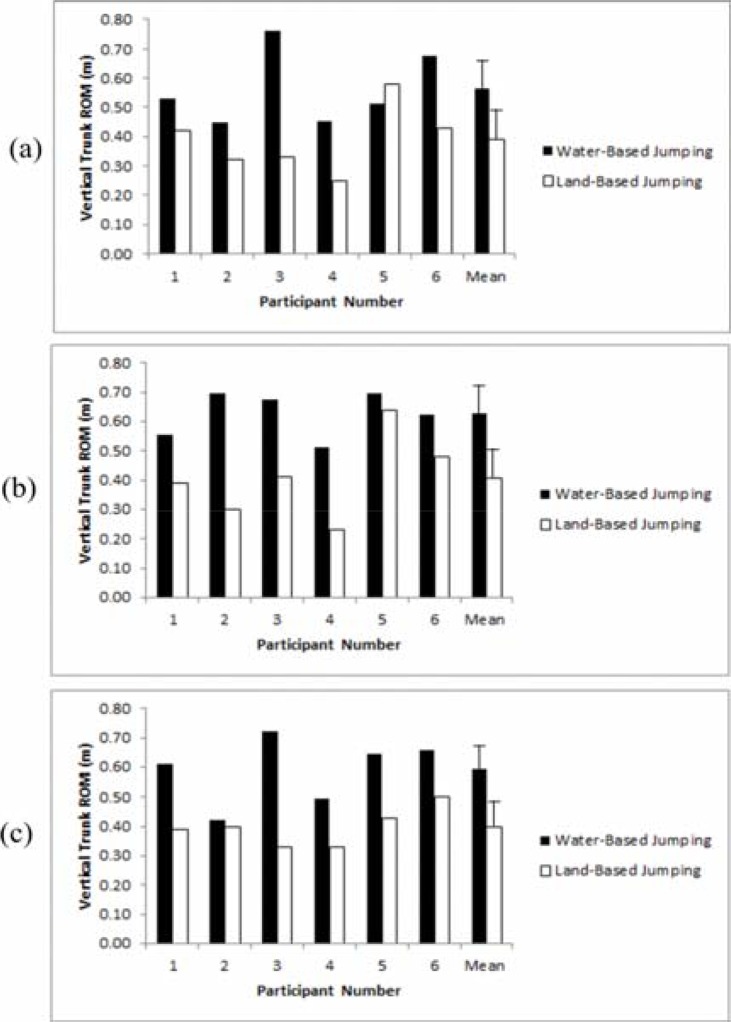
The vertical trunk ROM during water and land-based jumping in three breast supports (a) Bare-breasted, (b) Swimsuit, (c) Sports bra.

The mean vertical trunk ROM was 0.59 m in the water-based jumps and there was no significant difference in the vertical trunk ROM between breast support conditions (F=0.999, p=0.402, η^2^= 0.167). The mean vertical trunk ROM was 0.40 m in the land-based jumps with no significant differences in the vertical trunk ROM between breast support conditions (F=0.148, p=0.864, η^2^= 0.029). There was no significant difference in the vertical trunk ROM within the bare-breasted support condition between the water and land-based jumps (t=2.573, p=0.050), however, there were significant differences in the swimsuit support condition (t=4.394, p=0.007, d=1.9) and the sports bra support condition (t=3.999, p=0.010, d=2.1), with a greater trunk ROM during the water-based jumps (Figure 2).

### Breast kinematics during land-based jumping

During bare-breasted land-based jumping, a greater breast ROM was found in the superioinferior direction (0.095 m) compared to the mediolateral direction (0.052 m). The swimsuit (p=0.000, d=2.7) and the sports bra (p=0.001, d=3.8) were effective at significantly reducing the superioinferior breast ROM compared to bare-breasted jumping. The swimsuit (p=0.008, d=2.6) and the sports bra (p=0.022, d=1.9) were also effective at significantly reducing the mediolateral breast ROM compared to bare-breasted jumping (Table 1). Peak superioinferior breast velocity was greatest in the bare-breasted support condition (0.65 m/s), followed by the swimsuit (0.42 m/s) and the sports bra (0.32 m/s). There were significant differences between both the swimsuit (p=0.008, d=1.9) and the sports bra (p=0.005, d=2.9) compared to the bare-breasted condition. Peak mediolateral breast velocity was the greatest in the bare-breasted condition (0.46 m/s), followed by the sports bra (0.23 m/s) and the swimsuit (0.22 m/s). There were also significant differences between both the swimsuit (p=0.005, d=2.1) and the sports bra (p=0.007, d=2.0) compared to the bare-breasted condition (Table 1).

### Breast kinematics during water-based jumping

The superioinferior and mediolateral breast ROM were similar between breast supports (Table 2) and there were no significant differences in the amount of the superioinferior (F=0.335, p=0.723, η^2^=0.063) and mediolateral (F=5.211, p=0.071, η^2^=0.510) breast ROM, suggesting that neither the sports bra nor the swimsuit effectively reduced the breast ROM during water-based jumping. Peak superioinferior breast velocity was greatest in the swimsuit support condition (4.24 m/s), followed by the sports bra (3.90 m/s) and bare-breasted (3.78 m/s), however, there were no significant differences between support conditions (F=0.105, p= 0.901, η^2^=0.021). There were also no significant differences in peak mediolateral breast velocity (F=1.255, p=0.326, η^2^= 0.201), however the greatest peak mediolateral breast velocity was found in the swimsuit support condition (3.47 m/s), followed by the sports bra (2.21 m/s) and bare-breasted (1.60 m/s) (Table 2).

### Differences in breast kinematics between land and water-based jumping

The breast ROM and velocity were greater during water-based jumping compared to land-based jumping in all breast support conditions (p<0.05, d=1.7–4.2), with the exception of the superioinferior (t=1.934, p=0.111) and mediolateral (t=2.115, p=0.088) breast ROM in the bare-breasted condition.

### The breast water to air transition during water-based jumping

**Figure 3:**
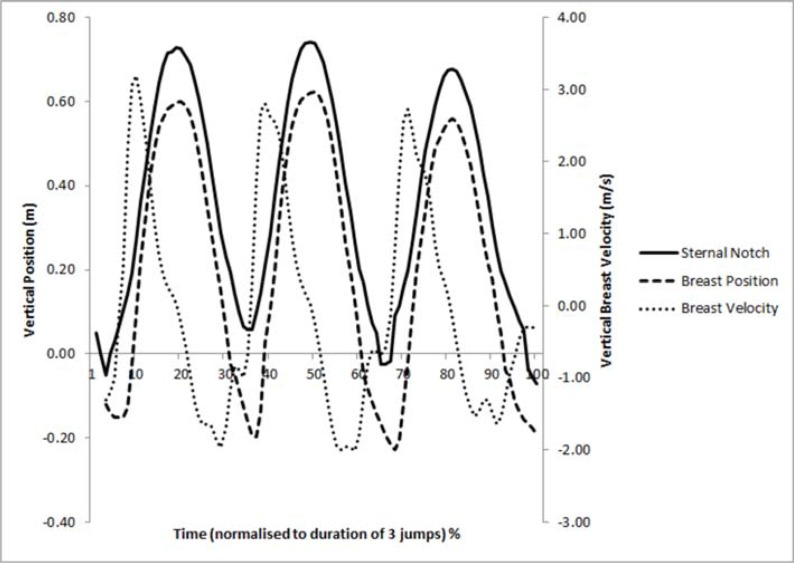
Examples of the position and velocity time history of the sternal notch and breast during jumping in water (n=1). Zero at the y axis equals water’s surface.

An interesting observation of the time normalised vertical trunk and breast position and breast velocity could help to explain the increased superioinferior breast velocity found during the water-based jumps. As the sternal notch (origin of the trunk) moved vertically higher during the jump, initially the breast remained in the water, as the breast approached the water’s surface the relative position between the nipple marker on the breast and the sternal notch marker increased, once the breast breached the water’s surface there was a rapid change in position (and hence velocity) as the breast ‘popped’ out of the water (Figure 3).

### Exercise Induced Breast Pain

Breast pain during land-based jumping (Table 1), bare-breasted (4 ±3), was reported as being twice that of breast pain experienced during water-based jumping (2 ±1) (Table 2), however, no significant differences (p>0.05) were found between breast pain in water-based when compared to land-based jumping in any of the support conditions. Furthermore, exercise induced breast pain was not significantly different between breast support conditions (Z=5.375, p=0.068) during water-based jumping, however, breast pain was significantly lower (Z=7.111, p=0.029) in the swimsuit condition during land-based jumping.

## Discussion

This preliminary study was the first to investigate the effect of breast support on breast kinematics and pain during land and water-based jumping. Key findings demonstrated that despite increases in the breast ROM and velocity, during water-based jumping compared to land-based jumping, there was no significant increase in exercise induced breast pain. This finding is contradictory to the previously published research on land that demonstrated an increased breast ROM and velocity were positively related to increased breast pain. Furthermore, the addition of an external breast support garment was able to reduce breast kinematics on land but not in water, suggesting the swimsuit and sports bra are not effective in terms of providing additional breast support during water-based jumping.

Previously published research has demonstrated that an addition of an external breast support garment, such as a bra, is capable of reducing breast kinematics whilst running (Scurr et al., 2009; 2010; White et al., 2009) and jumping (Bridgman et al., 2010) during physical exercise on land. Although the findings of this study also presented a significant reduction in breast kinematics with additional breast support on land (Table 1), a similar finding was not present during the water-based jumping activity, despite a similar trunk ROM (Table 2), partially rejecting hypothesis one. It was noted that during water-based jumping, the swimsuit was ‘bagging’ and therefore, it was not as tight around the breasts as it was on land. The ‘bagging’ caused water to become trapped in the upper section of the swimsuit, which reduced the support effectiveness of the garment and also influenced the movement of the breasts. These results suggest that the requirements of breast support garments, for larger breasted women, may be unique for water aerobics, which may offer a manufacturer the opportunity to develop a breast support garment biomechanically designed for this type of activity. Especially important to note is that the majority of breast displacement occurred as the breast exited and entered the water (Figure 3), this transition phase appears to place more demand on the breasts than current garments can support. Improving breast support garments for use in water, and making women aware of the importance of breast support, may decrease other negative effects associated with large magnitudes of breast motion such as embarrassment, a key barrier to physical activity participation (Burnett et al., 2014) and breast damage associated with skin strain causing breast ptosis (Silver et al., 2001). Findings from this study suggest further investigation into the support requirements of larger breasted women performing water-based exercises is required to improve breast support garments for this population group.

Greater breast kinematics were reported during water-based jumping compared to land-based jumping, rejecting hypothesis two. This is in conflict with the only water and land based breast kinematics research to date, which found a decrease in breast velocity during running in water when compared to land (McGhee et al., 2007). However, an increase in the trunk ROM was observed in the swimsuit and sports bra conditions during water-based jumping compared to the land-based jumping in the present study, this may have induced a greater magnitude of breast kinematics during water-based jumping as the trunk had been reported as the driving force for the breasts (Haake and Scurr, 2010). A further key factor that may have contributed to an increase in the breast ROM and velocity was associated with the breast’s transition from water to air during the water-based jump. Findings demonstrated (Figure 3) that at the start of the jump the trunk remained above the water’s surface with the breasts below. As the trunk moved higher during the jump the breasts remained submerged and the superioinferior displacement of the breast increased, then as the breast breached the surface of the water, the breast appeared to ‘pop’ out of the water causing a rapid change in position (velocity). The increased density of the water may restrict the motion of the breast whilst submerged, and ‘stretch’ the tissues of the skin and breast relative to the sternal notch, but as the breast breaks the surface of the water the breast recoils rapidly to catch up the trunk, thus increasing the breast ROM and peak breast velocity found in this study.

Increased exercise induced breast pain has previously been reported to be positively related to increases in breast displacement and velocity (Scurr et al., 2010), suggesting greater pain is experienced if the breast ROM increases, usually attributed to a lack of adequate breast support (Scurr et al., 2010; White et al., 2009). The findings of the present study showed that exercise induced breast pain did not significantly differ during water-based jumping compared to land-based jumping, rejecting hypothesis three. Furthermore, despite breast kinematics increasing from land-based to water-based jumping, no changes were evident in the breast pain experienced by the participants in this preliminary study. This suggests that a further mechanism may be responsible for the perceived pain previously attributed to increases in the breast ROM on land. It is possible that whilst the breasts are submerged in water they are in an equilibrium position where minimal tissue strain is being experienced. Subsequently as the breasts move during the water-based jump, they oscillate about this equilibrium position. Although the breast ROM may be greater in water-based jumping than on land-based jumping, the magnitude of the peak superior or inferior position may differ, as the breasts during land-based jumping are subjected to gravity and, therefore, already in a more inferior position at the start of the jump. This position may induce a pre-existing strain on the breast’s tissues; therefore, smaller increases in the magnitude of inferior breast motion may induce greater perceived breast pain. It is recommended that investigating the static and dynamic breast position in water and land may provide a better understanding of the possible sources of breast pain.

This preliminary study is the first to investigate breast kinematics and pain during water-based jumping. Key findings demonstrated that despite increases in the breast ROM and velocity during water-based jumping compared to land-based jumping, there was no increase in exercise induced breast pain. Furthermore, the additional breast support garments were ineffective at reducing breast kinematics during water-based jumping, suggesting that breast support design requirements may be unique for water aerobics, which may offer a manufacturer the opportunity to develop a breast support garment biomechanically designed for this increasingly popular type of activity. Improvements in swimwear breast support may help to reduce excessive breast motion during aqua aerobic jumping exercises.

## Figures and Tables

**Table 1:** The superioinferior (SI) and mediolateral (ML) breast ROM and peak velocity during land-based jumping in three breast support conditions

Breast support condition	Participant Number	Breast ROM (m)		Peak Breast Velocity (m/s)		Breast Pain

		SI	ML	SI	ML	
Bare-Breasted	1	0.100	0.046	0.73	0.49	6
	2	0.081	0.044	0.49	0.30	1
	3	0.107	0.079	0.81	0.72	6
	4	0.077	0.058	0.6	0.48	7
	5	0.108	0.049	0.73	0.43	1
	6	0.095	0.034	0.56	0.31	0
	Mean (SD)	0.095 (0.013)	0.052 (0.016)	0.65 (0.12)	0.46 (0.15)	4 (3)

Swimsuit	1	0.050	0.017	0.32	0.19	5
	2	0.037	0.021	0.28	0.13	0
	3	0.059	0.028	0.54	0.34	0
	4	0.043	0.020	0.36	0.24	1
	5	0.082	0.026	0.58	0.22	2
	6	0.061	0.020	0.44	0.19	0
	Mean (SD)	0.055 (0.016)	0.022 (0.004)	0.42 (0.12)	0.22 (0.07)	1 (2)

Sports Bra	1	0.026	0.028	0.23	0.23	3
	2	0.020	0.015	0.15	0.12	0
	3	0.049	0.031	0.37	0.38	0
	4	0.037	0.042	0.37	0.31	0
	5	0.052	0.011	0.38	0.10	0
	6	0.059	0.025	0.42	0.22	0
	Mean (SD)	0.041 (0.016)	0.026 (0.011)	0.32 (0.11)	0.23 (0.11)	1 (1)

**Table 2:** The superioinferior (SI) and mediolateral (ML) breast ROM and peak velocity during water-based jumping in three breast support conditions

Breast support condition	Participant Number	Breast ROM (m)		Peak Breast Velocity (m/s)		Breast Pain

		SI	ML	SI	ML	
Bare-Breasted	1	0.194	0.060	5.89	0.51	1
	2	0.064	0.047	2.70	1.03	3
	3	0.199	0.068	5.20	0.82	3
	4	0.091	0.120	2.20	2.21	2
	5	0.094	0.123	2.70	2.13	0
	6	0.200	0.154	4.01	2.90	0
	Mean (SD)	0.140 (0.064)	0.095 (0.043)	3.78 (1.51)	1.60 (0.94)	2 (1)

Swimsuit	1	0.115	0.044	3.80	6.73	0
	2	0.185	0.048	7.98	6.42	0
	3	0.127	0.063	3.40	0.81	1
	4	0.081	0.106	2.53	2.15	0
	5	0.130	0.127	4.37	1.92	1
	6	0.119	0.136	3.35	2.78	0
	Mean (SD)	0.126 (0.034)	0.087 (0.041)	4.24 (1.93)	3.47 (2.49)	0 (1)

Sports Bra	1	0.098	0.052	3.32	0.92	0
	2	0.052	0.048	1.98	0.88	0
	3	0.150	0.059	5.06	4.72	0
	4	0.098	0.105	4.00	2.09	0
	5	0.175	0.109	5.23	1.87	0
	6	0.130	0.126	3.80	2.75	0

	Mean (SD)	0.117 (0.044)	0.083 (0.034)	3.90 (1.20)	2.21 (1.43)	0 (0)

## References

[b1-jhk-46-49] Aquatic Exercise Association (2008). Aquatic fitness professional manual.

[b2-jhk-46-49] Ariyoshi M, Sonoda K, Nagata K, Mashima T, Zenmyo M, Paku C, Takamiya Y, Yoshimatsu H, Hirai Y, Yasunaga H, Akashi H, Imayama H, Shimokobe T, Innoue A, Mutoh Y (1999). Efficacy of aquatic-exercises for patients with low-back pain. Kurume Medical Journal.

[b3-jhk-46-49] Becker B (2009). Aquatic therapy: scientific foundations and clinical rehabilitation applications. Physical Medicine and Rehabilitation.

[b4-jhk-46-49] Benelli P, Colasanti F, Ditroilo M, Cuesta-Vargas A, Gatta G, Giacomini F, Lucertini F (2014). Physiological and biomechanical responses to walking underwater on a non-motorised treadmill: effects of different exercise intensities and depths in middle-aged healthy women. J Sport Sci.

[b5-jhk-46-49] Bridgman C, Scurr J, White J, Hedger W, Galbraith H (2010). Three-dimensional kinematics of the breast during a two-step star jump. J Appl Biomech.

[b6-jhk-46-49] Burnett E, White J, Scurr J (2014). The influence of the breast on physical activity participation in females. Journal of Physical Activity and Health.

[b7-jhk-46-49] Colado J, Tella V, Triplett N, Gonzales L (2009). Effects of short term aquatic resistance program on strength and body composition in fit young men. J Strength Cond Res.

[b8-jhk-46-49] Evans BW, Cureton KJ, Purvis JW (1978). Metabolic and circulatory responses to walking and jogging in water. Research Quarterly: American Alliance for Health, Physical Education and Recreation.

[b9-jhk-46-49] Field A (2009). Discovering statistics using SPSS.

[b10-jhk-46-49] Haake S, Scurr J (2010). A dynamic model of the breast during exercise. International Sports Engineering Association.

[b11-jhk-46-49] Kamalakkannan K, Vijayaragunathan N, Kalidasan R (2010). Analysis of aquatic and land training on selected physical fitness variables among volleyball players. Recent Research in Science and Technology.

[b12-jhk-46-49] Lorentzen D, Lawson L (1987). Selected sports bras: a biomechanical analysis of breast motion while jogging. The Physician and Sportsmedicine.

[b13-jhk-46-49] Mason B, Page K, Fallon J (1999). An analysis of movement and discomfort of the female breast during exercise and the effects of breast support in three cases. Journal of Science and Medicine in Sport.

[b14-jhk-46-49] McGhee DE, Power BM, Steele JR (2007). Does deep water running reduce exercise-induced breast discomfort?. Brit J Sport Med.

[b15-jhk-46-49] O’Donovan G, Blazevich A, Boreham C, Cooper A, Crank H, Ekelund U, Fox K, Gately P, Giles-Corti B, Gill J, Hamer M, McDermott I, Murphy M, Mutrie N, Reilly J, Saxton J, Stamatakis E (2010). The ABC of physical activity for health: a consensus statement from the British Association of Sport and Exercise Sciences. J Sport Sci.

[b16-jhk-46-49] Pendergast DR, Lundgren CEG (2009). The underwater environment: cardiopulmonary, thermal, and energetic demands. J Appl Physio.

[b17-jhk-46-49] Robertson CH, Engle CM, Bradley ME (1978). Lung volumes in man immersed to the neck: dilution and plethysmographic techniques. J Appl Physio.

[b18-jhk-46-49] Scurr J, White J, Hedger W (2009). Breast displacement in three dimensions during walking and running gait cycles. J Appl Biomech.

[b19-jhk-46-49] Scurr J, White J, Hedger W (2010). The effect of breast support on the kinematics of the breast during the running gait cycle. J Sport Sci.

[b20-jhk-46-49] Silver F, Freeman J, DeVore D (2001). Viscoelastic properties of human skin and processed dermis. Skin Res Tech.

[b21-jhk-46-49] Triplett N, Colado J, Benavent J, Alakhdar Y, Madera J, Gonzalez L, Tella V (2009). Concentric and impact forces of single-leg jumps in an aquatic environment versus land. Med Sci Sport Exer.

[b22-jhk-46-49] White J, Scurr J, Smith N (2009). The effect of breast support on kinetics during over-ground running performance. Ergonomics.

[b23-jhk-46-49] White J, Scurr J (2012). Evaluation of the bra fitting criteria for bra selection and fitting in the UK. Ergonomics.

[b24-jhk-46-49] Winter D (1990). Biomechanics and Motor Control of Human Movement.

[b25-jhk-46-49] Westby M (2001). A health professional’s guide to exercise prescription for people with arthritis: A review of aerobic fitness activities. Arthrit Care Res.

[b26-jhk-46-49] Zhou J, Yu W, Ng S-P (2012). Studies of three-dimensional trajectories of breast movement for better bra design. Text Res J.

